# ATP-Competitive MLKL Binders Have No Functional Impact on Necroptosis

**DOI:** 10.1371/journal.pone.0165983

**Published:** 2016-11-10

**Authors:** Bin Ma, Doug Marcotte, Murugan Paramasivam, Klaus Michelsen, Ti Wang, Andrea Bertolotti-Ciarlet, John Howard Jones, Ben Moree, Margaret Butko, Joshua Salafsky, Xin Sun, Timothy McKee, Laura F. Silvian

**Affiliations:** 1 Drug Discovery, Biogen Inc., Cambridge, MA, 02142, United States of America; 2 Biodesy Inc., South San Francisco, CA, 94080, United States of America; Toho Daigaku, JAPAN

## Abstract

MLKL is a pore forming pseudokinase involved in the final stage of necroptosis, a form of programmed cell death. Its phosphorylation by RIPK3 is necessary for triggering necroptosis but not for triggering apoptosis, which makes it a unique target for pharmacological inhibition to block necroptotic cell death. This mechanism has been described as playing a role in disease progression in neurodegenerative and inflammatory diseases. A type II kinase inhibitor (cpd 1) has been described that reportedly binds to the MLKL pseudokinase domain and prevents necroptosis. Here we describe five compounds that bind to the MLKL ATP-binding site, however the four MLKL-selective compounds have no activity in rescuing cells from necroptosis. We use kinase selectivity panels, crystallography and a new conformationally sensitive method of measuring protein conformational changes (SHG) to confirm that the one previously reported compound that can rescue cells (cpd 1) is a non-selective type II inhibitor that also inhibits the upstream kinase RIPK1. Although this compound can shift the GFE motif of the activation loop to an “out” position, the accessibility of the key residue Ser358 in the MLKL activation loop is not affected by compound binding to the MLKL active site. Our studies indicate that an ATP-pocket inhibitor of the MLKL pseudokinase domain does not have any impact on the necroptosis pathway, which is contrary to a previously reported study.

## Introduction

Necroptosis, a mechanism of caspase-independent, regulated cell death, has been described as part of a cell’s defense system against viruses [1]. Chronic inflammation underlies various neural and retinal degenerative diseases and the necroptosis pathway appears to be activated in cases where a receptor-associated complex fails to resolve the inflammatory condition. Unlike apoptosis, necroptosis does not involve caspase activation. The observation of necroptosis in various neurodegenerative pathologies like hypoxia-ischemia neuronal damage[2], ALS [3],[4], death of hippocampal neurons[5], retinitis pigmentosa[6], age-related macular degeneration[7], among others, makes inhibition of this form of cell death an attractive therapeutic strategy for neurodegenerative diseases[8].

The necroptosis pathway signals from TNF-alpha through a necrosome, consisting of RIPK1 and RIPK3 [9]. The necrosome forms in conditions where apoptosis is prevented, such as when c-IAPs are absent, translation is inhibited, TAK1 is inhibited, FADD has been knocked out, or RIPK1 is deubiquinated[10]. FADD (-/-) Jurkat cells have been engineered as an artificial cell model that bypasses apoptosis and where stimulation by TNF-alpha leads only to necroptosis [11].

**Fig 1:**
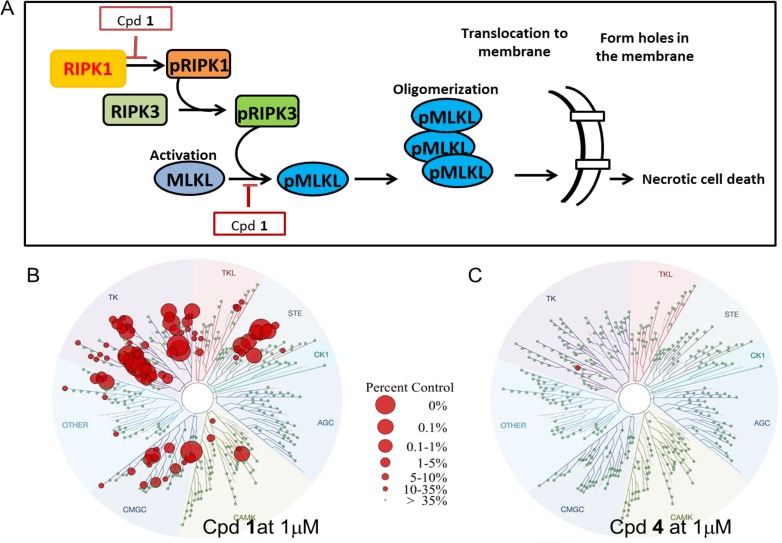
Necroptosis signaling pathway and selectivity of compounds in this study for one or more kinases with this pathway. (A) Necroptosis signaling pathway includes RIPK1, RIPK3 and MLKL (B) Analysis of cpd **1**% inhibition of 403 non-mutant kinases when tested at 1 μM concentration (the size of the red circle indicates a higher % inhibition). (C) Cpd **4** inhibits none of the 403 non-mutant kinases when tested at 1 μM concentration, but does inhibit pseudokinase MLKL.

The downstream effector of the necrosome is mixed lineage kinase like (MLKL) and its unique, discrete role in necroptosis makes it an attractive drug target (Fig 1). MLKL is a pseudokinase; it is structurally related to Ser/Thr kinases which bind ATP [12] but it has no ATPase or phosphotransfer activity, in part due a replacement of the DFG motif of normal kinases with a GFE motif in its activation loop and replacement of the catalytic loop residues HKD with an HGK motif. MLKL binds ATP in the absence of metal ions[12]. MLKL consists of an N-terminal 4-helix bundle domain linked to the C-terminal pseudokinase domain. RIPK3, activated within the necrosome, phosphorylates MLKL through its activation loop at Serine 358, which drives MLKL’s oligomerization and translocation to membranes, where it permeabilizes plasma membranes and other cellular organelles. While RIPK1 and RIPK3 are also involved in the apoptotic pathway, MLKL is unique to the necroptotic pathway.

The mechanism of signal transduction from the phosphorylated form of MLKL to oligomerization and pore-formation involving its N-terminal 4-helix bundle is not well understood. Since mutation of an activation loop residue to a phosphomimetic (S345D) and mutation of salt-bridged residues within the pseudokinase domain (K219M, Q343A) trigger necroptosis in the absence of stimuli, it has been argued that these mutations triggered a conformational change that unleashes the N-terminal pore-forming domain[12].

Kinase inhibitors can be categorized into several catagories, depending on whether they bind to the active conformation of the kinase domain. Type I kinase inhibitors form hydrogen-bonds with the kinase hinge and occupy the adenosine binding pocket. Type II kinase inhibitors occupy the adenosine pocket, but unlike type I binders, they induce a configuration of DFG residues termed “DFG-OUT”, in which the D of the DFG flipped 180° relative to the active state conformation. Often Type II compounds contain a benzamide portion, in which the benzene ring displaces the phenylalanine sidechain of the DFG motif[13].

Pharmacological inhibition of the necroptosis pathway by inhibiting either the RIP1 kinase active site or MLKL has been described in prior studies. Cpd **1,** which is a previously described, type II or “DFG-out” kinase inhibitor, has been identified in a previous high throughput screen for compounds that bind to MLKL[14] but which also is reported to bind to VEGFR2 and YES, among other kinases. The compound blocks necroptosis. The study suggested that the compound altered the conformation of the MLKL activation loop to make it more susceptible to RIPK3 phosphorylation. Secondly, Necrosulfonamide (NSA) has been previously identified as an inhibitor of MLKL, binding to cysteines within its N-terminal domain[15]. Lastly Necrostatin is a RIPK1-specific kinase inhibitor that protects cells against necroptosis but not apoptosis[16].

In this study we use methods such as thermal shift, x-ray crystallography and a new technique for measuring conformational changes—second harmonic generation—to characterize conformational changes in the pseudokinase and full length MLKL. For a couple of representative compounds we compare the induced conformational changes with overall specificity of kinase inhibition when tested against a broad panel. Lastly, we use a cell-based necroptosis assay to determine whether the compounds that bind MLKL indeed inhibit necroptosis, and if so, whether they block the accessibility of the MLKL activation loop towards phosphorylation by RIPK3.

## Materials and Methods

### Chemistry and Synthesis

Commercial reagents and solvents were used as received without further purification. Reactions were carried out under nitrogen in oven-dried glassware unless otherwise stated. ^1^H spectra were obtained using Bruker 400 MHz NMR spectrometers. LC-MS data were collected using a Micromass LCZ mass spectrometer with electrospray ionization in conjunction with a Waters 2795 LC system. Liquid chromatography (LC-MS) was performed using a SunFire ^TM^ C18, 3.5 μm, 4.6x20 mm IS ^TM^ column with mobile phase of 0.1% TFA in H_2_O (A) and 0.1% TFA in MeCN (B) and a gradient of 10–90% B in 2 min followed by 1 min at 90% B with flow rate at 3.0 ml/min. Detector was set at dual wavelengths 214 and 254 nm. Purity of compounds was also analyzed by RF-HPLC using the following two conditions:

(1) H_2_O/MeCN/0.1% TFA; (2) H_2_O/MeOH/0.1% TFA. The purity of all compounds listed were >95% at 254 nm. Analytical HPLC analysis was also carried out with a HP 1100 series using the same conditions. Preparative HPLC was carried out using a Gilson Platform equipped with UV/visible detector and an automatic fraction collector with SunFire ^TM^ C18, 5 μm, 19x150 mm OBD ^TM^ column and a gradient H_2_O/MeCN/0.1% TFA solvent system. Crizontinib (PF-02341066, Selleck Chemicals), cpd **1** (GW806742X, Sundia MediTech) and Necrosulfonamide (NSA; Toronto Research Chemicals) were used as provided. Cpds **2** and **3** were made following the literature precedent (WO2003066601)[17]. A racemic sample of cpd **4** and its enantiomer, cpd **5,** was provided by Enamine. The single enantiomer, cpd **4,** was prepared as described below.

#### Preparation of 3-Amino-pyrazine-2-carboxylic acid (R)-(4-fluorophenylcarbamoyl)-phenyl-methyl ester (cpd 4)

(R)-Hydroxy-phenyl-acetic acid (200 mg, 1 mmol) and p-fluoroaniline (146 mg, 1.31 mmol) in N,N-dimethylformamide (6.11 mL, 78.9 mmol) were added to HATU (0.600 g, 1.58 mmol) and N,N-diisopropylethylamine (0.916 mL, 5.26 mmol) and the mixture was stirred at room temperature overnight. After dilution with EtOAc and washing with water, brine, and drying over Na_2_SO_4_, the concentrated residue was purified by preparative HPLC to give product (R)-N-(4-Fluoro-phenyl)-2-hydroxy-2-phenyl-acetamide (174 mg, yield 50%). ESI-MS (M+H) ^+^: 246.1. ^1^H NMR (400 MHz, METHANOL-d4) δ 9.83 (br. s., 1H), 7.48–7.75 (m, 4H), 7.25–7.44 (m, 3H), 7.06 (t, J = 8.78 Hz, 2H), 5.16 (s, 1H).

Next, (R)-N-(4-Fluoro-phenyl)-2-hydroxy-2-phenyl-acetamide (140 mg, 0.57 mmol), 3-aminopyrazine-2-carboxylic acid (79.4 mg, 0.571 mmol), and 4-dimethylaminopyridine (34.9 mg, 0.285 mmol) in methylene chloride (2.20 mL, 34.2 mmol) were combined with N,N'-dicyclohexylcarbodiimide (118 mg, 0.571 mmol) in methylene chloride (1 mL, 20 mmol) at 0°C and the mixture was stirred for 3 hours. EtOAc was added and the resultant product filtered. The concentrated residue was purified with preparative HPLC to give compound **4** (105.9 mg, yield 51%). ESI-MS (M+H) ^+^: 367.1. ^1^H NMR (400 MHz, METHANOL-d4) δ 10.45 (s, 1H), 8.31 (s, 1H), 7.96 (s, 1H), 7.67 (d, J = 6.27 Hz, 2H), 7.58 (d, J = 4.77 Hz, 2H), 7.43 (d, J = 7.53 Hz,3H), 7.06 (t, J = 8.78 Hz, 2H), 6.36 (s, 1H).

### Necroptosis Assay

Necroptosis assays were performed at Charles River Laboratories (United Kingdom) and at Biogen. Briefly, FADD (-/-) Jurkat cells (ATCC #CRL-2572) were maintained in cell suspension in 1X RPMI medium 1640 (Gibco Life Technology Ref# A10491-01) supplemented with 10% heat inactivated FBS (Gibco Ref# 100820147) and 1X Penicillin/Streptomycin (from 100X stock solution, Corning Ref#30-002-CL) at 37°C, 5% CO_2_. On the day of the experiment, cells were counted using Nexcelom Bioscience Cell Meter Vision and pelleted by centrifugation. Cell pellets were resuspended in fresh medium at 500,000 cells per mL and transferred to assay-ready compound plates prepared in 384-well white TC plates (Greiner #781080) at 20μl cells per well. Cells and compound were incubated at room temperature for 30 minutes followed by +/- stimulation with 15ng/ml TNF-α (Cell signalling Technologies #8902LF) overnight at 37°C. In the morning, plates were equilibrated to room temperature for 30 minutes and 25μl of CellTiter-Glow (CellTiter-Glo 2.0 Assay, Promega # G9243) diluted 1:4 in PBS was added to each well and allowed to incubate for 10 minutes at room temperature. Luminescence was read on an Envision plate reader (Perkin Elmer).

TNF-α added to these cells induces necroptosis or cell death. The term “Rescue” refers to the ability of a compound to prevent loss of cell viability in the presence of TNF. Definitions of the presence or absence of compound or TNF stimulation in the wells that were used to calculate degree of rescue or viability is defined in Table 1.

**Table 1:** Definition of the terms used to calculate degree of rescue or viability, based on presence or absence of compound and TNF.

Term	Compound	TNF
“well”	+	+
“TNF”	-	+
“no TNF”	-	-
“Cpd”	+	-

**100% percent rescue** is defined as the value of the “no TNF” wells minus the value of wells treated with “TNF”.

**Percent Rescue** is then the ratio of the intensity value of the experimental “well” minus the value of “TNF” treated wells divided by the overall difference between the value of “no TNF” treated wells and the value of “TNF” treated wells.

**Percent viability** values are calculated from separate dose response experiments identical to the percent rescue experiment except that no TNF is added to the cells. This assay is intended to identify compounds that not only don’t rescue, but are toxic themselves.

**100% Viability** is defined as the intensity value of the “no TNF” wells, cell treated with neither compound nor TNF.

**Percent Viability** is the ratio of the intensity value of the wells that were treated with compound only (“cpd”) divided by the intensity value of wells treated with neither compound nor TNF (“no TNF”).

%rescue=((well - TNF)/(no TNF - TNF))x100

%viability=(cpd/no TNF)x100

### Mammalian Expression Constructs and MLKL Phosphorylation Assay (MSD)

The cDNA clones of human MLKL (RC213152) and human RIPK3 (RC209549) were purchased from Origene. To generate GFP-MLKL and RIPK3-mCherry, coding sequences of the corresponding cDNA sequences were PCR amplified and cloned into Biogen’s mammalian expression vector.

293T cells grown in 10 cm plates were transfected with GFP-MLKL alone or both GFP-MLKL and RIPK3-mCherry plasmids. After 24 hours, cells were harvested and replated into poly D lysine coated 96W plate with 20,000 cells/ml. Four hours later, cells were treated with MLKL compounds for 30 min and incubated at 37°C. Thirty minutes later, the media was removed and cells were lysed in 150μl of Cell lysis buffer (Cell Signaling, MA) containing both protease and phosphatase inhibitors. The lysates were transferred to 96 well MSD plates (Meso Scale Discovery, MD) that were precoated with anti-mouse MLKL antibody H00197259-M02, 3B2, Novus Biologicals, CO), and incubated overnight at 4°C. After washing the plates three times with 1x MSD wash buffer (50mM TRIS-HCl, 150mM NaCl, 0.02% Tween 20, pH7.5), 100 μl of 0.5μg/ml of rabbit-anti-MLKL phospho Ser358 (Abcam AB187091) was added to each well. The plates were incubated for 2 hours at room temperature. After three washes with MSD wash buffer, 100 μ l of 0.5ug/ml of SULFO-tag labeled anti-rabbit IgG was added to each well and incubated for 1 hour at room temperature. After three washes, 1x Read bufferT (Meso Scale Discovery, MD) was added and the electrochemiluminescence (ECL) was measured with a MSD Sector Imager 600.

### Competition Binding Assay

Compound binding constants (K_D_s) were determined at DiscoverX using their KINOMEscan assay platform. This competition binding assay was previously described in Karaman et al. (2008)[18]. Briefly, full length MLKL was expressed in HEK-293 cells as a fusion with an NFκB DNA binding domain and subsequently tagged with a qPCR detection amplicon via interaction with NFκB. Binding reactions consisted of tagged MLKL extract, active site competitive probe attached to affinity beads, and test compound. Twenty microliters of binding reactions were carried out in 384-well polypropylene plates for 1 hour at room temperature. Beads were subsequently washed and resuspended in elution buffer for 30 minutes at room temperature with shaking. The concentration of MLKL in the eluates was measured by qPCR and compound binding constants were calculated using a standard dose-response curve.

### Production of Recombinant MLKL Constructs

#### Pseudokinase domain constructs (MLKL-1 and MLKL-2)

MLKL-1 was constructed by sub-cloning residues 191 to 471 of the pseudokinase domain of human MLKL cDNA into a pET15b vector with a thrombin cleavable N-terminal six histidine fusion tag (His-MLKL-1). MLKL-2 had the same protein boundaries and fusion tag system as MLKL-1 but the dual mutations E366A and K367A were introduced using site directed mutagenesis and was expressed in the same vector (His-MLKL-2). These residues were identified as having high surface entropy and were mutated to alanine to improve crystal packing.

BL21 (DE3) *Escherichia coli* cells were transformed with the plasmids encoding either pseudokinase protein construct and were grown at 37°C in LB media supplemented with ampicillin to an OD of 0.6. The temperature was reduced to 18°C and protein expression was induced by adding 0.1mM IPTG and shaking for an additional 16 hours. The cells were harvested and resuspended in lysis buffer (50mM TRIS pH 8.0, 500mM NaCl, 10% Glycerol, 1mM DTT and Roche EDTA-free protease inhibitor cocktail) and were lysed using a microfluidizer. The lysate was clarified by centrifugation at 20,000 x g for 1 hour at 4°C and the His-MLKL was captured by batch binding to Nickel NTA resin overnight at 4°C. The resin was washed with buffer A (25mM TRIS pH 8.0, 250mM NaCl, 10% glycerol, 1mM DTT) and loaded onto a XK column and washed until the absorbance at 280nm dropped to baseline. His6-MLKL1 or His-MLKL2 was eluted from the column using buffer A supplemented with 250mM imidazole pH 8.0 and analyzed by SDS-PAGE.

His-MLKL-1 protein was further purified using a Superdex 75 column equilibrated in buffer B (25mM TRIS pH 8.0, 250mM NaCl, 5% glycerol and 2mM DTT) and it eluted as a monomer. To achieve untagged protein, the his-tagged fusion was treated with Thrombin Protease (10units/mg of protein) while dialyzing against buffer C (25mM TRIS pH 8.0, 250mM NaCl, 5% glycerol, 1mM CaCl_2_ and 1mM DTT) for 18 hours at 4°C. The His6 liberated MLKL-2 protein was then rerun over Nickel NTA and the flow through was collected and concentrated for further purification on a Superdex 75 column equilibrated in buffer B (25mM TRIS pH 8.0, 250mM NaCl, 5% glycerol and 2mM DTT). MLKL-2 also eluted as a monomer.

#### Full length human MLKL (MLKL-3)

The full length MLKL was prepared in baculovirus using a bacmid DNA which was modified to include a Tev cleavable N-terminal His_6_ maltose binding protein (His-MBP) tag. His-MBP-MLKL-3 was expressed in baculovirus-infected sf9 insect cells grown in SF-900 II serum-free medium (Invitrogen). Cells were harvested by centrifugation 48 hours post-infection. The cells were resuspended in lysis buffer (50mM TRIS pH 8.0, 500mM NaCl, 10% glycerol, 1mM DTT and Roche EDTA-free protease inhibitor cocktail) and were lysed using a microfluidizer. The lysate was clarified by centrifugation at 20,000 x g for 1 hour at 4°C and the His-MBP-MLKL-3 was captured by batch binding to Nickel NTA resin overnight at 4°C. The resin was washed with buffer A (25mM TRIS pH 8.0, 250mM NaCl, 10% glycerol, 1mM DTT) and loaded into an XK column and washed until no non-specific unbound protein was detected. His-MBP-MLKL3 was eluted from the column using buffer A supplemented with 250mM imidazole pH 8.0 and analyzed by SDS-PAGE. Fractions containing His-MBP-MLKL-3 were pooled and dialyzed against 25mM MES pH 6.0, 50mM NaCl, 5% glycerol and 2mM DTT for 18 hours at 4°C and then loaded onto a Mono-S column which was washed and eluted with a salt gradient. The His-MBP-MLKL-3 eluted at approximately 250mM NaCl.

### Second Harmonic Generation Experiments

#### Lipids and SLB Preparation

The Ni-NTA bilayer surface is available from Biodesy (South San Francisco, CA). Preparation of the bilayer surface on glass was performed according to manufacturer instructions.

#### Protein Labeling

The labeling reaction was performed according to manufacturer’s instructions. Briefly, His-MLKL-1 and His-MBP-MLKL-3 proteins were exchanged into 100mM NaH_2_PO_4_, pH 7.5, 150mM NaCl, 0.5mM TCEP prior to labeling with a 7K MWCO Zeba desalting column (Thermofisher). For the labeling reaction, a 5-fold molar excess of the second-harmonic-active dye SHG1-SE (available from Biodesy) was added to the protein solution and the reaction was allowed to proceed for 10 minutes at room temperature. The samples were then centrifuged for 20 minutes at 16.8K*g and the supernatant passed through a Zeba desalting column to remove free dye from the reaction. UV-Vis spectrophotometry was used to determine the degree of labeling.

#### Mass Spectrometry Analysis

LC-MS/MS was performed by Martin Protean, LLC (Princeton, NJ) as a fee for service. Samples were denatured, reduced, and alkylated by diluting 10μL of MLKL protein into a solution containing 60μL of a 7M Guanidinium Chloride, 3μL 1M Na_x_H_y_PO_4_ pH 7.4, 0.5μL 2M dithiothreitol, and 1.5μL 1M iodoacetamide and incubated at room temperature for 30 minutes. Samples were then TCA precipitated by adding 300μL 0.025% sodium deoxycholate and 30μL 50% trichloroacetic acid followed by centrifugation for 10 minutes at 16k*g in an Eppendorf 5415D centrifuge at room temperature. The supernatent was removed and the sample was washed twice with 200μL acetone and centrifuged for 3 minutes at 16k*g at room temperature. The precipitated protein was then resuspended in 20μL 8M Urea buffered with 200mM Hepes pH 8.0 and then immediately diluted with 60μL freshly prepared 0.1mg/mL Chymotrypsin & Trypsin buffered in 100mM Hepes pH 8.0, 5mM CaCl2. Proteolysis was allowed to proceed at room temperature for 30 minutes. To remove the proteases, 320μL of acetone was added to the samples, followed by centrifugation for 10 minutes at 16k*g at room temperature. The supernatant was then transferred to a glass vial and the acetone was allowed to evaporate overnight.

Liquid Chromatography Tandem Mass Spectrometery Liquid chromatography was performed using a nanoAcquity ultra performance LC system (Waters, Milford, MA, USA). Mass Spectrometry was performed using a Deca XP+ Iontrap Mass Sepctrometer, (Thermofisher Scientific, San Jose, CA, USA). Four microliters of sample were injected onto a trapping column, washed at room temperature for 3 minutes at 15μL per minute. After buffer salts were removed, peptides were resolved over a 75μm C18 BEH nanoAcquity column using a gradient from a 1‐41% acetonitrile‐0.1% formic acid against water with 0.1% formic acid, over 20 minutes, at 0.6μL/min. The mass spectrometer was set in the NSI configuration in the positive ion mode for LC/MS/MS experiments, with the spray voltage set to 1.7kV and the ion transfer capillary temperature set to 150°C. The ionization was further tuned automatically using the m/z ratio of 445. The first round of mass spectrometry data was collected scanning from 400‐2000 m/z. The second round of tandem mass spectrometry was performed by automation, with the most intense ion in the first round being selected for subsequent fragmentation in the ion trap by helium bombardment.

Mass spectrometry analysis revealed that full-length MLKL-3 was modified at residue K230 (peptide APVAIK*VF; M/Z = 607.1; 18.8 minute retention time) and residue K255 (peptide KK*FESPNILR, M/Z = 552.6; 11.7 minute retention time). Analysis of the relative abundance of the total modified peptides indicated that 93% of the total modified peptides had SHG dye attached at residue K230 and the remaining 7% of modified peptides had attached dye at residue K255.

Mass spectrometry analysis of the pseudokinase domain (MLKL-1) indicated that the protein was modified at residue K219 (peptide YK*GEY, M/Z = 514.4; 15.1 minute retention time) and residue K230 (peptide APVAIK*VF; M/Z = 607.0; 18.7 minute retention time). Analysis of the relative abundance of the total modified peptides of the MLKL pseudokinase domain indicated that 32% of the total modified peptides were modified at residue K219 and the remaining 68% of modified peptides had SHG dye attached at residue K230.

#### Sample Preparation for SHG

Bilayer formation and protein deposition was performed as previously described[19] except for the following changes. A custom 384 well microtiter plate (Biodesy Delta Read Plates) was used to form supported lipid bilayers for protein deposition. A Bravo automated liquid-handling platform (Agilent) was used for bilayer deposition, buffer washes, and protein deposition during sample preparation.

For MLKL experiments, the assay buffer was 20mM HEPES, pH 8.0, 200mM NaCl, 0.005% Tween-20. MLKL protein was deposited at a final concentration of 1μM in each well and allowed to incubate on the Ni-NTA surface overnight at 4°C. After overnight incubation, unbound protein was removed by washing with assay buffer plus 1% DMSO. Plates were then allowed to equilibrate for 30 minutes at room temperature before beginning compound injections.

#### SHG Instrumentation and Experiments

Experiments were performed on a Biodesy Delta System. The Biodesy Delta system is a 384-well microplate-based instrument, combining optical detection of the SHG signal with automated liquid handling to enable detection of conformation change in real-time, synchronized with ligand injection. Delta’s optical subsystem comprises a femtosecond titanium sapphire (Ti:S) laser as the source of the fundamental beam at 800 nm. The fundamental is directed to an arrangement of prisms integrated with the custom, glass-bottomed Biodesy Delta Read Plates, where it undergoes total internal reflection (TIR) to generate the evanescent field at the glass-lipid bilayer interface. This evanescent field interacts with the SHG-active probe attached to the bilayer-tethered protein, generating the second-harmonic (SH) light at 400 nm, which emerges from the interface in a collimated beam nearly collinear with the fundamental beam. The fundamental light is filtered out leaving only the SH light which is detected by a photomultiplier tube and processed with custom electronics. The SHG signal is the intensity of the SH light (photons/s). The liquid handling subsystem is designed to enable high-throughput, automated measurements using a standard 384-well microplate format. Ligand injection into the read plate is accomplished with an on-board 8-channel MEMS-based electronic pipetting system, enabling high speed and accuracy. The system is controlled by a web-based user interface, enabling the user to visually design complex experiments using multiple ligand plate. The software displays results in real time, numerically or graphically, and allows data to be exported for further analysis.

#### SHG Quantification

To plot the percent change in SHG intensity (%Δ_SH_) over time, the second-harmonic baseline intensity measured just prior to injection (I_t0_) was subtracted from the second-harmonic intensity at each time interval (I_tn_) and then divided by the initial second-harmonic intensity (I_t0_) according to Eq 1:

$$\%\Delta_{\text{SH}}=\left({\text{I}}_{\text{tn}}\;\text{-}\;{\text{I}}_{\text{t}0}\right){/\text{I}}_{\text{t}0}$$ (1)

In addition, all experiments included a control buffer injection that was used to determine the significant threshold for SHG intensity change and was calculated in a similar manner. All values in the paper are reported as the mean ± SE of the percent change for each independent experiment.

#### Data Analysis

The normalized SHG intensity values were calculated as indicated above, and were imported into GraphPad Prism. The data were plotted as XY plot with the Mean ± SEM reported for each point on the kinetic curve.

### Differential Scanning Fluorimetry of MLKL Pseudokinase, RIPK1 Kinase and HSP90 N-Terminal Domains

To measure the thermal stabilization of the proteins upon compound binding we employed differential scanning fluorimetry (DSF). The kinase domain of RIPK1 and the N-terminal domain HSP90α were expressed and purified as previously described [20][21]. The pseudokinase MLKL-1, RIPK1 and Hsp90 were melted at 1μM final concentrations in 25mM HEPES pH 8.0, 250mM NaCl and 2.5mM DTT. Compounds were added to a final concentration of 10 μ M with a final DMSO concentration of 5%. The complexes were incubated on ice for 30 minutes. Protein Thermal Shift dye (Life Technologies) was added to 1X. The fluorescence intensities were measured using a Quantstudio real-time PCR system (Life Technologies) with excitation at 470 nm and emission at 586 nm. The samples, measured in triplicate, were heated from 25 to 85°C with a heating rate of 0.017°C per min.

### Crystallization and Structure Determination

The complex of untagged MLKL-2 and cpd **1** was formed using 5mg/ml protein and two fold molar excess of cpd **1** and incubated on ice for 1 hour. Crystals of the MLKL-2/cpd **1** complex were grown in 0.1 Sodium Citrate and 15% PEG3350 at 18°C. The crystals were transferred to a cryoprotection solution containing 0.1 Sodium Citrate, 15% PEG3350 and 15% Glycerol and flash frozen in liquid nitrogen prior to data collection.

The MLKL-2/Cpd **4** structure was produced through soaking experiments. APO MLKL-2 crystals were grown using 5mg/ml protein in 0.1M BisTRIS pH 6.5 and 25% PEG300 at 18°C. The crystals were then soaked for 16 hours at 18°C with 1mM cpd **4**. The soaked crystals were then transferred to a cryoprotection solution containing 0.1M BisTRIS pH 6.5 and 35% PEG300 flash frozen in liquid nitrogen prior to data collection.

X-ray diffraction data was collected at LRL-Cat at the Argonne Photon Source and was processed using Mosflm[22] or XDS[23]. Both data sets were solved using molecular replacement using Phaser[24] [24]using the human MLKL pseudo kinase domain structure (PDB ID: 4MWI)[25] as the search model. Multiple rounds of refinement using REFMAC5.0[26] and subsequent model building using COOT[27] led to models for the MLKL-2/cpd **1** and MLKL-2/cpd **4** structures with an Rfree/ R of at 26.4%/ 19.9% to 2.88 Å resolution and 21.7%/ 18.7% to 2.16 Å resolution, respectively, both with good geometry. The structure of MLKL-2/cpd **1** has been deposited with PDB ID 5KNJ and MLKL-2/cpd **4** has been deposited with PDB ID 5KO1 (S1 Table).

## Results

### Identification of Compounds Displacing an ATP-Competitive Probe in MLKL

To identify compounds that specifically bind the active site of MLKL we used an ATP-competitive probe displacement assay (DiscoverX). Established compounds were also tested in this assay and characterized by their binding affinities to MLKL. A similar approach was used to measure binding affinities for RIPK1 and RIPK3 kinase domains.

**Fig 2:**
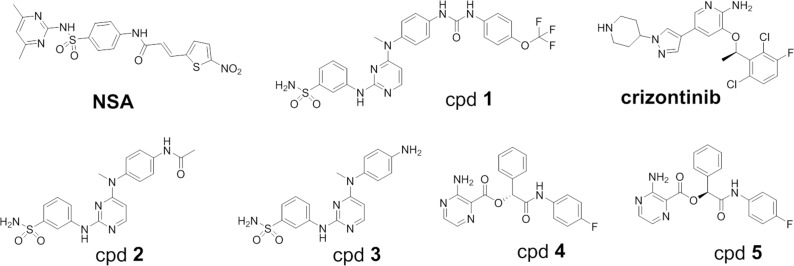
Chemical structure of MLKL binders described in text.

Using the displacement assay as a high throughput screening (HTS) assay, a 5000 diversified compound library was screened for compounds that bind to MLKL but not to RIPK1 or RIPK3. While 173 compounds bound to MLKL with >80% displacement potency at 10μM, only a handful of compounds were selective when counterscreened on RIPK1 and RIPK3. A racemic mixture of cpds **4, 5** (Fig 2) was originally identified, followed by enantiomeric separation. Cpd **4** (MLKL K_D_ = 230 nM) binds selectively to MLKL without binding RIPK1 or RIPK3 (Table 2). Its enantiomer (cpd **5**) had weak MLKL binding affinity (MLKL K_D_ = 7600 nM) (Table 2).

**Table 2:** Compound binding affinities for MLKL, RIPK1 and RIPK3, differential thermal shift values and Necroptosis Inhibition.

Compound	MLKL Displacement KD (nM)*	RIPK1 Displacement KD (nM)*	RIPK3 Displacement KD (nM)*	RIPK1 kinase thermal shift at 10 μM (°C)^	MLKL Pseudo-kinase thermal shift at 10 μM (°C)^	Necroptosis cell IC50 (μM)*
**NSA**	-	> 30,000	> 30,000	0.15	0.67	0.2
**Crizontinib**	217	1,300	6,700	0.75	5.63	>40
**cpd 1**	116	370	750	5.86	5.75	1.85
**cpd 2**	780	> 10,000	-	0	2.93	>40
**cpd 3**	880	> 10,000	-	0.31	1.57	>40
**cpd 4**	230	> 30,000	> 30,000	-0.15	4.13	>40
**cpd 5**	7600	> 30,000	> 30,000	-	-	-

*average of duplicates

^average of triplicates

We then characterized several compounds that were expected to bind to MLKL across a broad panel of 403 non-mutant kinases at DiscoverX. Cpd **1**, previously identified by Hildebrand et al.[14], was found to be highly nonselective in which 56 out of 403 kinases were inhibited at 1μM (Fig 1B), while cpd **4** was found to be highly selective, hitting only 1 pseudokinase (MLKL) out of the 403 collective targets at 1μM concentration (Fig 1C). Necrosufonaminde (NSA) was found not to displace the ATP probe from MLKL, RIPK1, or RIPK3, which is consistent with the finding that the compound binds to a cysteine in the N-terminal 4-helix bundle domain[15] (data not shown). **Crizotinib** was identified as binding with higher affinity to MLKL than to RIPK1 or RIPK3 (K_D_ = 210 nM for MLKL) (Table 2). Cpd **1** (Fig 2) was found to bind to both MLKL and RIPK1 with 116 and 370 nM K_Ds_ respectively. Truncated versions of cpd **1** in which the urea phenyl-OF3 group was removed (cpd **2,** cpd **3**) (Fig 2) were expected to bind as type I inhibitors since we removed the terminal phenyl group that would have otherwise displace the MLKL Phe 350 from its pocket. The truncated analogues were found to bind to MLKL (K_D_ = 780nM and 880nM respectively) with at least 10-fold weaker potency to RIPK1 (Table 2).

### Necroptosis Assay

**Fig 3:**
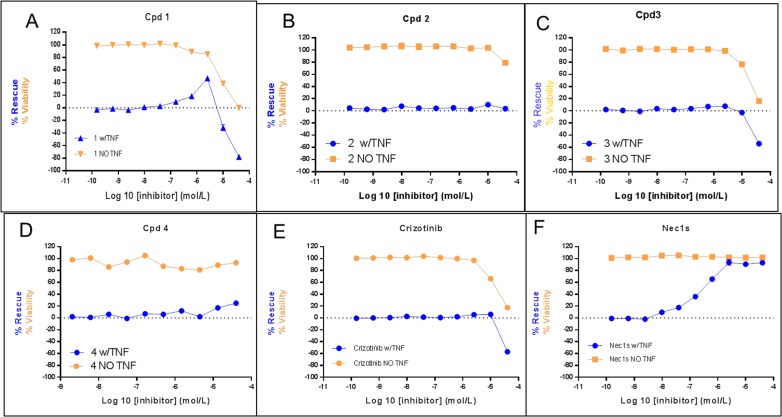
Necroptosis assay using FADD-deficient Jurkat cells measuring compound-dependent rescue from cell death. The different compounds described in text were tested for rescue in dose-response curves: (A) cpd **1** (B) cpd **2** (C) cpd **3** (D) cpd **4** (E) **Crizotinib** (F) Necrostatin (Nec1s). The blue line represents the rescue experiment from necroptosis in the presence of TNF and the yellow line represents the viability experiment in the absence of TNF.

The identified MLKL-specific and non-specific ATP-competitive compounds were next tested for inhibition of necroptosis in the FADD deficient Jurkat cell necroptosis assay. The positive control compound (Nec1s) rescued cells as described in the literature with EC50 = 0.5 μM. The compound does not effect cell viability in the absence of TNF stimulation (Fig 3F). Similarly, cpd **1** rescued at an EC50 of 1.85 μM, but cell viability became an issue at > ~2μM compound concentrations (Fig 3A). Interestingly, neither the MLKL selective analog cpd **4** (Fig 3D) nor the truncated versions of cpd **1** with better selectivity against RIPK1 (cpds **2,3**) (Fig 3B and 3C) rescued cells from necroptosis. Similarly, **Crizotinib**, which also binds to MLKL does not rescue cells from necroptosis (Fig 3E). Our preliminary hypothesis was that type I compounds are unable to alter the activation loop of MLKL to protect it from phosphorylation by RIPK3, while type II compounds are able to cause a conformational change that reduced the activation loop accessibility and prevent MLKL’s phosphorylation.

### Thermal Shift to Measure Stabilization of Proteins

We next wanted to test whether these compounds could be differentiated by how they stabilized the protein against thermal denaturation. We used a thermal denaturation assay to test whether there are functional differences in the ability of type I and type II compounds to stabilize the protein structure at an inhibitor concentration of 10 μM. Our data suggests that all compounds that bind with triple digit nM activity stabilize MLKL similarly, whether they are type I or type II. Serving as positive controls, we confirmed that necrostatin (Nec1s) prefers RIPK1 and that **Crizotinib** prefers MLKL (S2 Table). In comparison, necrosulfonamide (NSA) stabilizes neither the RIPK1 kinase nor the MLKL pseudokinase domain, as expected, due to its binding to the N-terminal 4-helix bundle (Table 2). Cpd **1** stabilizes both MLKL and RIPK1 (thermal shift of +5–6°C at 10μM compound concentrations relative to the apo protein), whereas cpds **2, 3**, and **4** stabilize MLKL over RIPK1; the most potent compound (cpd **4** binding to MLKL) specifically leads to a thermal shift of +4°C. However, the extent of thermal stabilization of the MLKL pseudokinase domain for a type I (cpd **4)** and type II (cpd **1**) compound was similar. Our data indicates that both type I and type II inhibitors can improve the stability of MLKL in a similar fashion, and is in general correspondence with the KDs measured from the ATP-competitive probe displacement assay.

### Biodesy Conformational Assay Differentiates Binding Modes

Since we did not observe any difference in the thermal stability between type I and type II compounds, we wished to confirm that binding of the compound would induce a conformational change in the protein. To further confirm our expectations of the binding mode of the ATP mimetic MLKL binders as type I or type II, we developed a conformational assay with Biodesy using their Second Harmonic Generation (SHG) technology.

The SHG signal depends on the dye-labeled protein’s physical orientation relative to the surface. SHG is highly sensitive and can detect changes as small as ~1 degree of probe angular change, and it is mass independent with no size or mass constraints[19,28–30]. Both pseudokinase His-MLKL-1 and full length His-MBP-MLKL-3 were rendered second harmonic active by labeling lysine residues with an amine reactive second harmonic dye (Biodesy, SHG1-SE). Mass spectrometry analysis revealed that full-length His-MBP-MLKL-3 was modified primarily at residue K230 (93%) and the remainder at residue K255 (7%) (data not shown). The His-MLKL-1 pseudokinase domain was also modified primarily at residue K230 (68%) and the remainder at residue K219 (32%)(data not shown).

**Fig 4:**
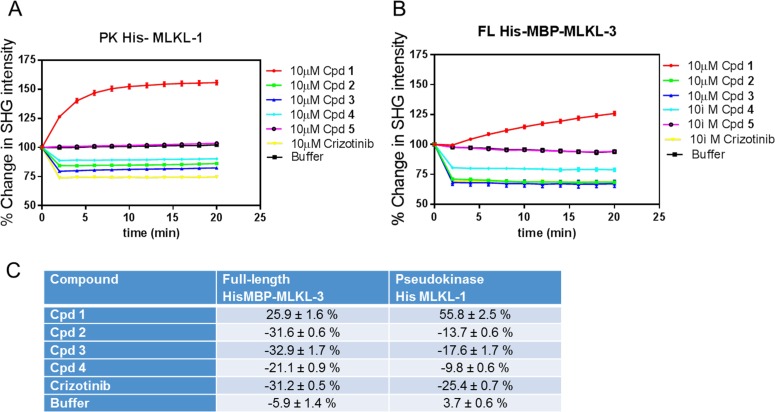
Changes in SHG intensity over time upon compound binding. (A) full length (FL) MLKL and (B) pseudokinase (PK) domain SHG intensity with 10 μM compound addition. (C) Quantitation of % change overall for the kinetic curves.

After labeling, each compound was tested for its ability to modulate the conformation of MLKL by measuring the change in the SHG signal upon compound binding. Each compound was added at 10μM final concentration and the change in SHG intensity was measured over a period of 20-minutes. Incubation of cpd **1** with full length MBP-MLKL resulted in a 25.9 ± 1.6% increase in the SHG intensity of the labeled, surface-tethered protein (Fig 4A). Interestingly, incubation of cpds **2, 3, 4** and **Crizotinib** resulted in an overall net decrease in the SHG intensity (Fig 4C). Similar results were also obtained for the MLKL pseudokinase domain (Fig 4B). Given the differences in the change in the SHG intensity between this series of compounds, the data suggest that MLKL may exist in a similar overall conformation when bound to type I compounds and that this conformation is structurally distinct from the conformation of the type II cpd **1** bound MLKL.

In addition to measuring the overall change in the SHG intensity, the kinetic time course of the change in SHG intensity of each compound was also measured. Addition of cpd **1** to the full-length MBP-MLKL protein resulted in a gradual increase in the overall SHG intensity over the course of the experiment (Fig 4A). This is in contrast to the results obtained with cpds **2, 3, 4, and Crizotinib** in which a rapid and stable, step-like decrease in the SHG intensity is observed. Experiments with the MLKL pseudokinase domain produced similar results (Fig 4B). The inactive cpd **5** resulted in no change from baseline and overlays with the buffer control. These results indicate that the binding of cpds **2,3,4** and **Crizotinib** to MLKL produce conformational changes in MLKL that occur on a faster timescale than those with cpd **1**. Taken together, these SHG data further support the conclusion that the mechanism of action and resulting conformational changes in MLKL differ between the two sets of compounds, and is consistent with their expected characterization as type I or type II (“GFE-out”compounds) in which the compound must displace the phenylalanine sidechain from its pocket.

### Xray Co-Crystal Structures

**Fig 5:**
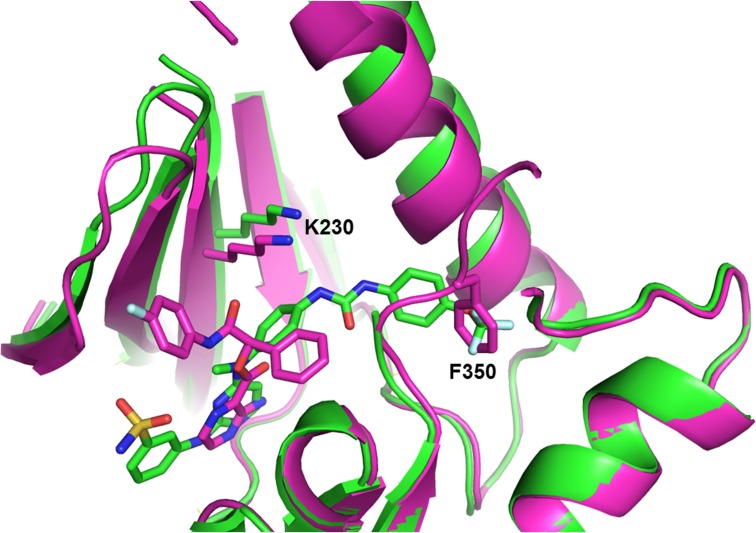
Superposition of the crystal structures of MLKL pseudokinase bound to cpd 1 (green) or cpd 4 (magenta). The C-terminal domain of both structures were superimposed. Phe 350 of the GFE motif in hMLKL is highlighted to demonstrate the conformational changes induced by binding the type II cpd **1**. The catalytic lysine (K230) which is labeled by the SHG-sensitive dye is also highlighted.

We then wanted to confirm the binding modes for cpd **1** and cpd **4** to the pseudokinase domain of MLKL by crystallography. To this end, we co-crystallized and solved the structure of cpd **1** and cpd **4** with the MLKL pseudokinase domain to a resolution of 2.88 Å and 2.16 Å, respectively (Fig 5). As expected from the SHG studies, the structures confirm that cpd **1** is a type II inhibitor and cpd **4** is a type I inhibitor.

The co-crystal structure showed that cpd **1** binds in the ATP binding site of MLKL by making two hydrogen bond interactions between the backbone amide and carbonyl of the hinge and the amino pyrimidine of the compound (Fig 5, green). There is also one hydrogen bond with Glu293 of MLKL and the sulfonamide of the compound. The urea linker hydrogen bonds to Glu250 of the c-helix and backbone amide of Gly349 orients the trifluoromethoxyphenyl into the pocket where the Phe 350 sidechain of the GFE motif within the APO structure would reside, confirming type II binding. Residues 350–369 of the activation loop are missing in the cpd **1** cocrystal structure due to disorder.

The 2.1 Å co-crystal structure of cpd **4** confirms that it is a type I MLKL binder. The co-crystal structure shows that cpd **4** binds in the ATP binding site of MLKL making two hydrogen bonds with the hinge and several hydrogen bonds with two nearby water molecules (Fig 5, magenta). The 4-fluoro-phenyl is oriented toward the P-loop and stacks with the aliphatic segment of the Arg210 sidechain which covers the ATP binding pocket. The non-substituted phenyl is pointing toward the GFE motif of MLKL but does not displace Phe350 from its pocket, consistent with type I binding. While the GFE sequence is ordered, residues 355–368 of the activation loop are disordered in the cpd **4** structure.

The crystal structures shed light on the observed difference in binding kinetics between type I and type II compounds. The K230 side chain, which is labeled by the SHG dye, moves 2.8 Å in the type II structure (green) relative to its position in the type I co-crystal structure (magenta), when the C-terminal domains are aligned, due to general movement of the N-terminal domain relative to the C-terminal domain (Fig 5). Domain-domain motion can take minutes to come to equilibrium. This could explain the differences observed in SHG intensity and kinetics for a type I and type II compounds.

### Compounds Do Not Prevent RIPK3 Phosphorylation of MLKL

**Fig 6:**
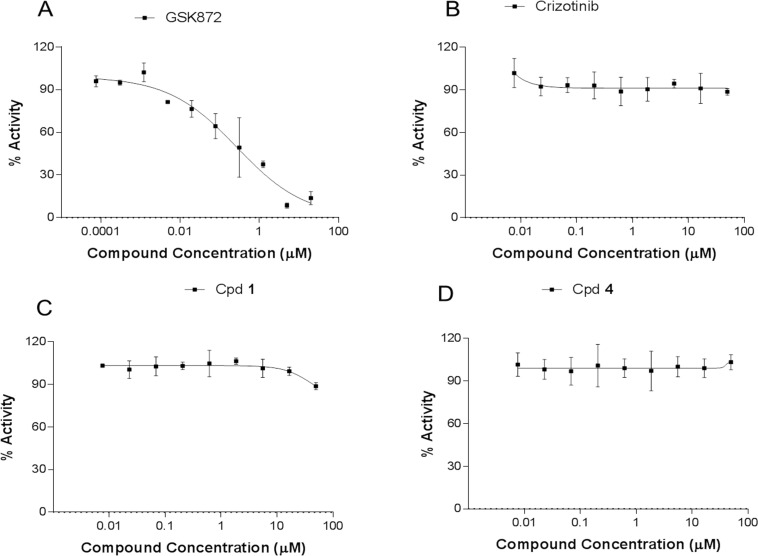
MSD assay monitoring RIPK3-dependent phosphorylation of the MLKL activation loop. (A) GSK872 compound that specifically inhibits RIPK3 inhibits phosphorylation of MLKL as positive control. (B) Crizotinib binding to MLKL does not impact its phosphorylation by RIPK3. (C) Cpd **1** binding to MLKL does not impact its phosphorylation by Ripk3. (D) Cpd **4** binding to MLKL does not impact its phosphorylation by RipK3. Data is normalized against a reaction in the presence of DMSO alone (100% activity).

Having tool compounds that bind to MLKL allowed us to determine whether they might restrict MLKL’s activation loop accessibility and prevent its phosphorylation by RIPK3. We coexpressed RIPK3 and MLKL In 293T cells to generate a phosphorylated MLKL sample that could be detected with an anti-phospho-MLKL antibody. In order to demonstrate that the assay was dependent on RIPK3 phosphorylation of MLKL, we demonstrated that the phosphoserine detection signal was completely inhibited by incubation of cells with a RIPK3-specific compound GSK compound (GSK872, Calbiochem). We found that neither cpd **1** or its truncated type I analogue (cpd **3**) when incubated with these cells had any significant impact on the phosphor MLKL levels (Fig 6). This contrasts with the claim made in Hildebrand et al., 2014[14] that cpd **1** enhanced phosphorylation of the MLKL activation loop. This disproves our preliminary hypothesis that type II compounds binding to MLKL would have an effect on the accessibility of the activation loop and thus prevent necroptosis.

### Testing Cpd 1 Binding to Hsp90

A recent report suggests that Hsp90 can modulate the stability of the RIPK3/MLKL complex and the oligomerization of MLKL and that an Hsp90 inhibitor can protect against Necroptosis[31]. To confirm that cpd **1** is not acting through Hsp90 inhibition, we compared the thermal stability of the Hsp90α catalytic domain bound to a positive control compound 17-AAG vs. cpd **1**. (S2 Table). Addition of control compound (17-AAG) to Hsp90 at 10μM produced a +3.3°C shift, while the same concentration of cpd **1** only produced a +1.5°C shift.

## Discussion

Our expectation in designing ATP-displacing compounds that are MLKL-specific was that they would either protect its activation loop from being phosphorylated or reduce the conformational changes that MLKL could adopt that lead to pore formation. It has been reported that phosphorylation of Ser358 of human MLKL triggers further translocation, oligomerization and pore formation of MLKL[9]. There has been only one compound described to date that binds the ATP pocket of MLKL and has an effect on Necroptosis (cpd **1**) by Hildebrand et al[14]. We wished to better understand its mechanism of action to design similar compounds.

In this study we categorized compounds as type I or type II using a novel, conformationally sensitive method employing second harmonic generation (SHG) technology. An SHG-sensitive probe was attached to the catalytic lysine of the MLKL active site. We noted that the probe labeling does not interfere with binding affinity of the compound through surface plasmon resonance studies (data not shown).

Both the direction of the SHG signal intensity and the kinetics of binding are important variables in distinguishing the two binding modes. The type II binder bound to the pseudokinase domain and caused the SHG intensity to increase over the course of 5 minutes. As the intensity of the SHG signal is dependent on the angle between the SHG dye and the surface normal, an increase in SHG intensity as seen with the type II binder indicates that the dye has moved toward the surface normal, i.e more perpendicular to the surface plane, upon compound binding. The same compound, upon binding to the full length MLKL tethered to the plate at a different site, also caused the same slow movement of the dye, but in this case the rate of conformational change is slower and did not reach equilibrium within the > 20 minutes observation window. In contrast, the binding of type I compounds causes the SHG signal to decrease in intensity, indicating that the orientation of the dye has moved away from the surface normal and become more parallel to the surface plane. We conclude that SHG thus presents a useful method for distinguishing type I and type II binding modes without the complexities of crystal structures.

The type I and type II co-crystal structures of human MLKL differ not only in the relative orientation of the N- and C-terminal domains but also in the conformation of the GFE region of the activation loop. The remaining part of the activation loop, including the Ser 358 (site of phosphorylation by RIPK3), is disordered in both structures. Compared to the APO human MLKL structure (PDBID 4MWI), the type I compound-bound structure shows very little differences overall, with conformational changes in the P-loop only. In contrast, the type II structure differs significantly from the APO structure—namely the GFE which marks the beginning of the activation loop shifts and guides this part of the activation loop in a different direction.

Surprisingly, we saw no real difference in the phosphorylation state of MLKL when a type I or type II compound was bound after 30 minutes of incubation with the transiently transfected 293 cells overexpressing RIPK3 and MLKL. The compound had no effect on the accessibility of the MLKL substrate in this instance. It remains to be determined whether a specific type II compound binding to MLKL active site could effect the accessibility of the activation loop, as we were unable to identify a type II compound that specifically bound MLKL. Alternatively, an inhibitor that somehow specifically concealed Ser358 in the MLKL activation loop would be an alternative way to block necroptosis.

MLKL is a relatively unique target for necroptosis, which differentiates it from RIPK1 and RIPK3 which are common to other forms of cell death and signaling. When we profiled the kinases that cpd **1** inhibits, we found that not only does it inhibit the kinases VEGFR2 and YES as described in the original report, but also hits upstream kinase RIPK1, which could explain its ability to rescue cells from Necroptosis. This mechanism of action has precedence—a different compound identified by Degterev et al. that specifically inhibits the RIPK1 kinase active site (Nec1) also protects cells from Necroptosis but not Apoptosis[16]. Cpd1 also appears to not impact apoptosis, based on studies carried out by Hildebrand et al., consistent with the notion described in a recent paper by Suda et al. contrasting the effect of knockdown of RIPK1 compared to the D168N mutant form of RIPK1, which lacks kinase activity, on apoptosis[32]. Alternatively, cpd **1** might also bind weakly to Hsp90 and reduce the chaperone’s ability to form the RIPK1/RIPK3 necrosome or oligomerized MLKL. In conclusion, our revised hypothesis is that the effect of cpd **1** on Necroposis is likely due to polypharmacology resulting from its non-specific binding to kinases such as RIPK1, rather than its specific effect on MLKL. Taken together with data from our selective MLKL type I binders, we propose that the binding of compounds to the ATP-pocket of the MLKL pseudokinase domain alone will not rescue cells from necroptosis.

## Supporting Information

S1 Table Data collection and refinement statistics for MLKL pseudokinase bound the cpd 1 and cpd 4. (TIF)

S2 Table Thermal shift stabilization upon binding compounds to catalytic domain of MLKL, RIPK1, Hsp90α. (TIF)

## Abbreviations

*MLKL*: Mixed lineage kinase domain-like
*FADD*: Fas-associated protein via a death domain
NSA: Necrosulfonamide
*RIPK*: Receptor-interacting protein kinase
RHIM: RIP homotypic interaction motif
