# Supplementary figures and images for: ATP-Competitive MLKL Binders Have No Functional Impact on Necroptosis
Source: PLoS One. 2016 Nov 10;11(11):e0165983. doi: 10.1371/journal.pone.0165983 (PMC5104457; doi:10.1371/journal.pone.0165983)

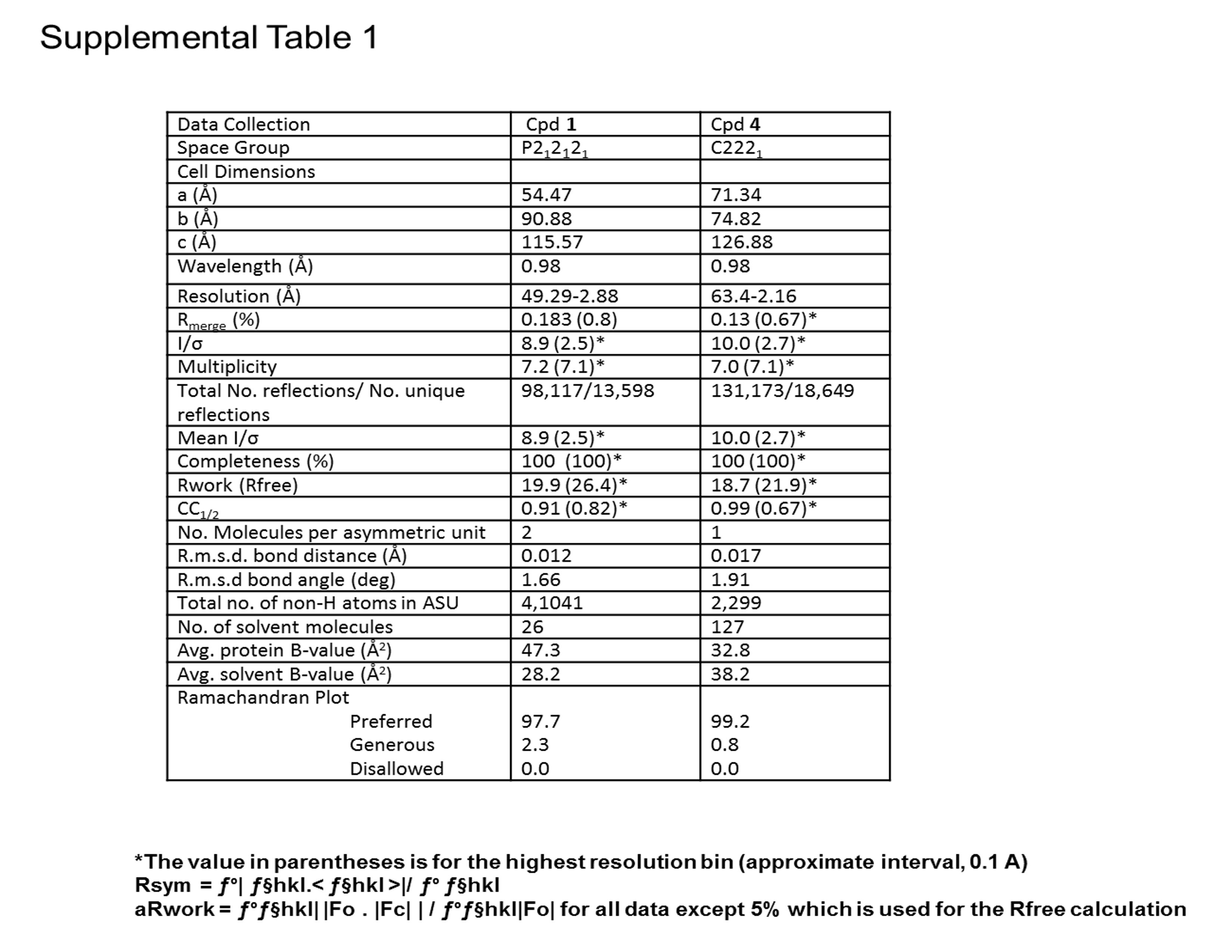

Supplement: S1 Table — (TIF) [file pone.0165983.s001.tif]

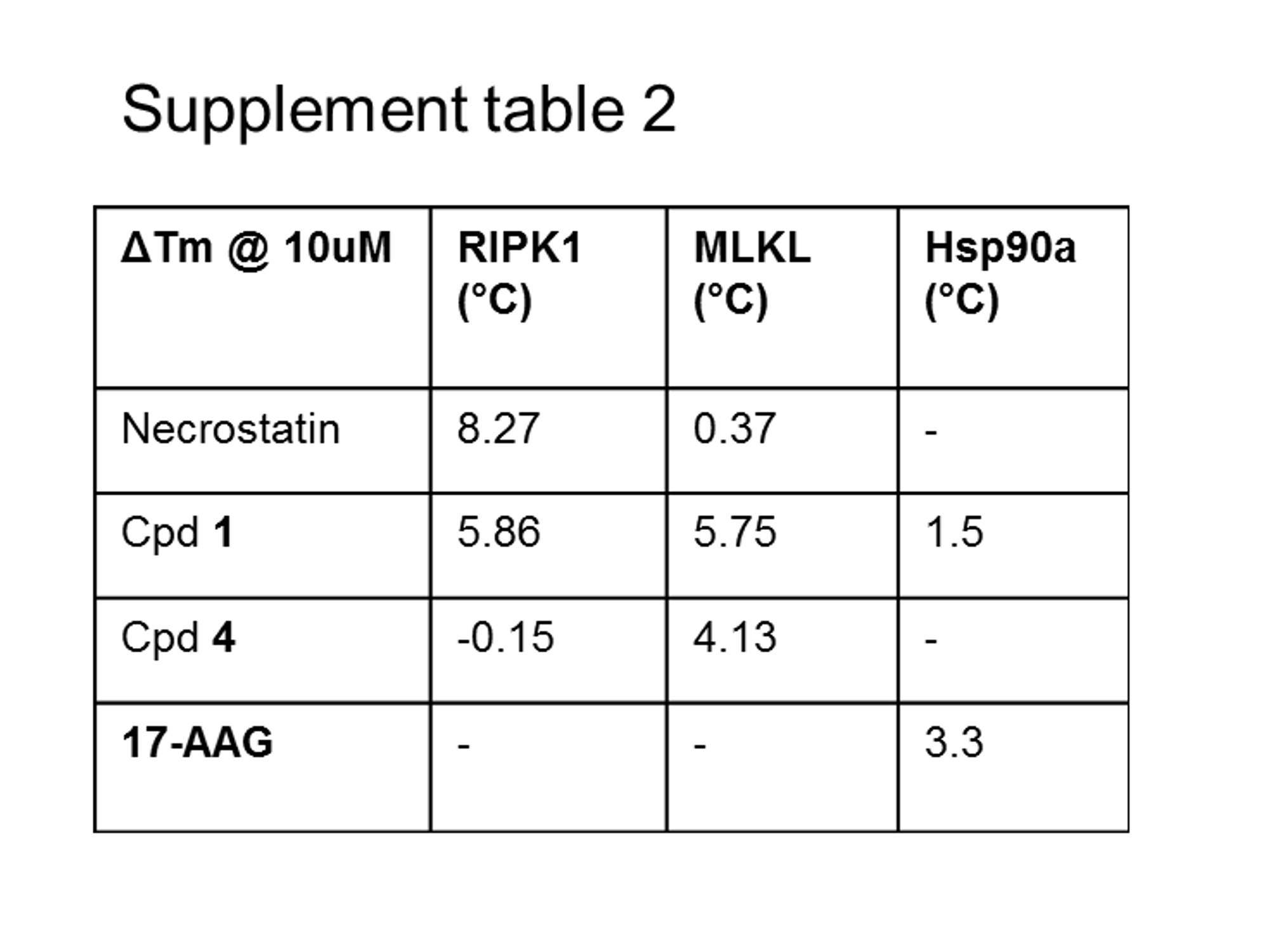

Supplement: S2 Table — (TIF) [file pone.0165983.s002.tif]
